# Laminectomy for Spinal Injuries

**Published:** 1894-03

**Authors:** 


### LAMINECTOMY FOR SPINAL INJURIES.

Mr. T. Myles read notes of a case of laminectomy for spinal injuries, before the Royal Academy of Medicine in Ireland, at the meeting of December 8, 1893. The patient was sixty-five years old, and had fallen backward from a donkey cart, striking the ground with the back of his head and neck, so that his chin was driven on to his chest with some violence. Paraplegia ensued, and an operation was decided upon. Incision was made from a little below the seventh cervical vertebra, the muscles were cleared, and the vertebral arches exposed; it was then found that several of them were shattered and their fragments pressing on the cord. The loose fragments were first removed, and then the arches of the fourth, fifth and sixth vertebræ were completely detached by cutting forceps and the dura exposed. As this seemed tense, it was opened and the cord examined; no blood could be seen, and the cord seemed healthy so far as it was visible from the wound. The patient experienced some slight relief for a few hours after the operation but died next day, three days after the accident. On making a *post mortem* examination it was found that, in addition to the fracture of the vertebral arches already alluded to, there was also present a fracture involving the body of the third cervical vertebra. The cord, when examined from the front, had the appearpearance of being slightly bruised, but was by no means in the shattered condition anticipated from the severity of his symptoms. There was very little blood at the seat of fracture, and no displacement of fragments from the body of the vertebra.

Mr. Myles offered the following propositions:

1. That in certain cases of spinal fracture an operation may be attempted with reasonable hope of benefit.

2. That those cases are alone likely to be successfully treated by operation in which the cord is not completely crushed or divided.

3. That the existence of motor paralysis alone is not proof of hopeless injury to the cord.

4. That the persistence of sensation shows that the cord is not hopelessly injured, even though motor palsy be present.

5. That the complete, permanent absence of all reflex phenomena is proof that the cord is hopelessly damaged.

6. That where the symptoms are caused by indirect violence applied to the spine, as a general rule, operation is not to be thought of.

7. That where the symptoms are caused by direct violence the propriety of operating depends upon the severity of the injuries and accompanying symptoms.

8. That when the injury is caused by direct violence, if the reflexes persist and if sensory impressions are still appreciated, an operation may be attempted with reasonable hopes of benefiting our patients.

9. That when the symptoms are due to pressure from extravasated blood, operation is to be advised.

10. That the terrible fate awaiting most patients suffering from severe spinal injury justifies even the most hazardous operations, if any hope of benefit therefrom can be reasonably entertained.

Dr. F. A. Nixon said that the difficulties in such cases was to make out accurately to what extent the spine was injured by the original accident. Apart from that, the great severity of the operation, which required deep incisions and the removal of the laminæ, does not seem to favor surgical interference.

Dr. Doyle had operated on a child five and a half years old, suffering from paralysis, increased tendon reflexes, rigid muscles and cystitis. After removing portions of the vertebræ there was improvement for a few days, but this did not continue. Suspecting an abscess formation compressing the cord, he had no difficulty in letting out the abscess, but experienced a great deal in keeping it open. The child died, six weeks later, from cystitis and renal trouble.

Dr. Kendal Franks thought that Mr. Myles was perfectly justified in performing the operation, and that he acted properly in removing the long sections pressing on the spinal cord, but that the dura mater should not have been opened unless absolutely necessary.

Mr. Myles replied that the dura mater seemed, both to his colleagues and to himself and to Dr. Nixon, more prominent than it ought to be, and when pressed upon it gave a sense of fluctuation ; and, as the phenomena outside was insufficient to account for the paralysis, it was resolved to puncture, less there might be any possible compression from blood, etc.—*Medical Press and Circular* January 3, 1894 (*Ex*).

